# Decoding human cancer with whole genome sequencing: a review of PCAWG Project studies published in February 2020

**DOI:** 10.1007/s10555-021-09969-z

**Published:** 2021-06-07

**Authors:** Simona Giunta

**Affiliations:** 1 Laboratory of Genome Evolution, Department of Biology & Biotechnology “Charles Darwin”, University of Rome Sapienza, Rome, Italy; 2 The Rockefeller University, 1230 York Avenue, New York, NY USA

**Keywords:** Cancer, Genomes, Whole genome sequencing, Pan-Cancer project, PCAWG, Driver mutations, Chromothripsis, Telomeres, RNA

## Abstract

Cancer is underlined by genetic changes. In an unprecedented international effort, the Pan-Cancer Analysis of Whole Genomes (PCAWG) of the International Cancer Genome Consortium (ICGC) and The Cancer Genome Atlas (TCGA) sequenced the tumors of over two thousand five hundred patients across 38 different cancer types, as well as the corresponding healthy tissue, with the aim of identifying genome-wide mutations exclusively found in cancer and uncovering new genetic changes that drive tumor formation. What set this project apart from earlier efforts is the use of whole genome sequencing (WGS) that enabled to explore alterations beyond the coding DNA, into cancer’s non-coding genome. WGS of the entire cohort allowed to tease apart driving mutations that initiate and support carcinogenesis from passenger mutations that do not play an overt role in the disease. At least one causative mutation was found in 95% of all cancers, with many tumors showing an average of 5 driver mutations. The PCAWG Project also assessed the transcriptional output altered in cancer and rebuilt the evolutionary history of each tumor showing that initial driver mutations can occur years if not decades prior to a diagnosis. Here, I provide a concise review of the Pan-Cancer Project papers published on February 2020, along with key computational tools and the digital framework generated as part of the project. This represents an historic effort by hundreds of international collaborators, which provides a comprehensive understanding of cancer genetics, with publicly available data and resources representing a treasure trove of information to advance cancer research for years to come.

**Supplementary Information:**

The online version contains supplementary material available at 10.1007/s10555-021-09969-z.

## Introduction

Cancer is the second most frequent cause of death worldwide. A “cure” for cancer has long been sought, yet cancer represents a very heterogeneous group of diseases sharing a few defining phenotypic characteristics at the cellular level, such as unregulated proliferation. Cancer genomes encapsulate multiple genetic complexities that are often deeply intertwined, including (1) heterogeneity across populations and individuals, (2) cancer evolution that promotes genetic diversity, and (2) age-related increase in mutational burden. The PCAWG Project sampled patients between 1 and 90 years old in its large cohort, estimating ~ 190 single nucleotide changes suffered by the genome on a daily basis.

Mutations can be inherited, called germline mutations, or somatic – which are randomly acquired during a person’s lifetime. The PCAWG Project investigated both types of these variations in cancer cells and identified the genetic changes that have a causal role in cancer.

**Fig. 1:**
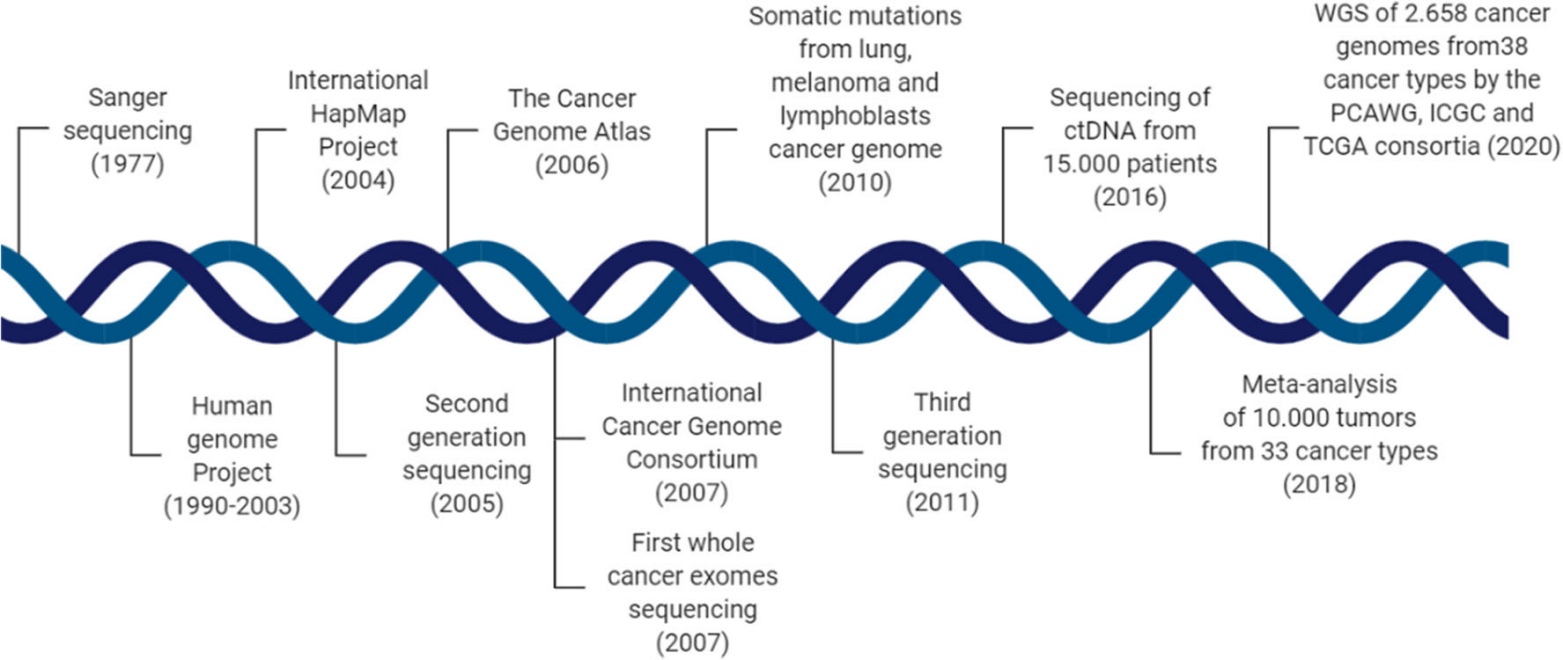
Key advances in understanding cancer genomes. A timeline of key technological advances in sequencing, seminal milestones and large-cohort studies published in the last 50 years (not to scale) that have contributed to our current understanding of mutations driving cancer.

The finding, detailed in an impressive collection of 23 papers published in *Nature* and affiliated journals accessible on the Nature Pan-Cancer Analysis of Whole Genomes page (nature.com/collections/afdejfafdb), builds upon earlier efforts by TCGA established by the NIH (USA) and the ICGC. The complete set of samples from over 2,600 patients across 38 different types of cancers of the PCAWG Project represents the most comprehensive study of whole cancer genomes to date (Fig. 1).

Here, I offer a concise review of key findings that have emerged from this seminal work (Table 1), with each chapter proving a snapshot for each of the main studies. I also review the methodologies and computational tools, datasets, and digital framework generated by the PCAWG consortium (Table 2), with a description of the publications dedicated to software development and computational advances available as Supplemental Material. Readers are encouraged to read the original publications and refer to original and official sources cited throughout this review, especially cancer research scientists and clinicians, as aspects of these pivotal studies are hereby omitted due to space constraints.

**Table 1:** PCAWG Project major findings reviewed here. The published collection of papers can be accessed on the Nature website landing page for the PCAWG Consortium (www.nature.com/collections/afdejfafdb).

Publication	Brief description	Chapter	Reference
Campbell PJ, Getz G, Korbel JO, Stuart JM, Jennings JL, Stein LD, et al. Pan-cancer analysis of whole genomes. Nature. 2020 10.1038/s41586-020-1969-6	Identified driver mutations across cancer genomes	3.1	[2] Preprint [75]
Rheinbay E, Nielsen MM, Abascal F, Wala JA, Shapira O, Tiao G, et al. Analyses of non-coding somatic drivers in 2,658 cancer whole genomes. Nature. 2020 10.1038/s41586-020-1965-x	Analysis of the 13% of tumor samples that have non-coding mutations that drive cancer	3.2	[10] Preprint [76]
Alexandrov LB, Kim J, Haradhvala NJ, Huang MN, Tian Ng AW, Wu Y, et al. The repertoire of mutational signatures in human cancer. Nature. 2020 10.1038/s41586-020-1943-3	Identified new signatures of mutational processes that cause base substitutions, small insertions and deletions, and structural variation in cancer	3.3	[15] Preprint [77]
Li Y, Roberts ND, Wala JA, Shapira O, Schumacher SE, Kumar K, et al. Patterns of somatic structural variation in human cancer genomes. Nature. 2020 10.1038/s41586-019-1913-9	Identified new signatures of mutational processes that cause larger-scale structural variations associated with cancer	3.3	[16] Preprint [78]
Gerstung M, Jolly C, Leshchiner I, Dentro SC, Gonzalez S, Rosebrock D, et al. The evolutionary history of 2,658 cancers. Nature. 2020 10.1038/s41586-019-1907-7	Analysis of the timings and mutational patterns in the evolution of tumors to map the progression and occurrence of each driver	3.4	[29] Preprint [79]
Calabrese C, Davidson NR, Demircioğlu D, Fonseca NA, He Y, Kahles A, et al. Genomic basis for RNA alterations in cancer. Nature. 2020 10.1038/s41586-020-1970-0	Describes the diverse transcriptional consequences of somatic mutation on splicing, expression levels, fusion genes, and promoter activity	3.5.1	[22] Preprint [80]
Zhang Y, Chen F, Fonseca NA, He Y, Fujita M, Nakagawa H, et al. High-coverage whole-genome analysis of 1220 cancers reveals hundreds of genes deregulated by rearrangement-mediated cis-regulatory alterations. Nature Communications. 2020 10.1038/s41467-019-13885-w	Analysis of the diverse transcriptional consequences of gene deregulation by non-coding regions in cancer	3.5.2	[33] Preprint [81]
Rodriguez-Martin B, Alvarez EG, Baez-Ortega A, Zamora J, Supek F, Demeulemeester J, et al. Pan-cancer analysis of whole genomes identifies driver rearrangements promoted by LINE-1 retrotransposition. Nature Genetics. 2020 https://doi. org/10.1038/s41588-019-0562-0	Evaluates “jumping” of retrotransposable elements as a driver of cancer-associated mutagenesis	3.6	[24] Preprint [82]
Sieverling L, Hong C, Koser SD, Ginsbach P, Kleinheinz K, Hutter B, et al. Genomic footprints of activated telomere maintenance mechanisms in cancer. Nature communications 10.1038/s41467-019-13824-9	Explores different known and still unknown pathways used by cancer to maintain their telomeres	3.7	[5] Preprint [83]
Yuan Y, Ju YS, Kim Y, Li J, Wang Y, Yoon CJ, et al. Comprehensive molecular characterization of mitochondrial genomes in human cancers. Nature Genetics. 2020 10.1038/s41588-019-0557-x	Mutational analysis of mitochondrial DNA in cancer	3.8	[49] Preprint [84]
Akdemir KC, Le VT, Sahaana C, Li Y, Group P-SVW, Verhaak RG, et al. Chromatin Folding Domains Disruptions by Somatic Genomic Rearrangements in Human Cancers. Nat Genet. 2019 10.1038/s41588-019- 0564-y	Alterations of 3D genome architecture in cancer	3.9	[23] Preprint [85]
Reyna MA, Haan D, Paczkowska M, Verbeke LPC, Vazquez M, Kahraman A, et al. Pathway and network analysis of more than 2500 whole cancer genomes. Nature Communications. 2020 10.1038/s41467-020-14351-8	Establishes the most commonly mutated pathways and molecular processes in driving cancer formation and progression	3.10	[52] Preprint [86]
Cortés-Ciriano I, Lee JJK, Xi R, Jain D, Jung YL, Yang L, et al. Comprehensive analysis of chromothripsis in 2,658 human cancers using whole-genome sequencing. Nature Genetics. 2020 10.1038/s41588-019-0576-7	Analysis of chromothripsis, a mutational process found to occur early in a high proportion of all cancers and to drive tumor genetic heterogeneity	3.11	[25] Preprint [87]
Zapatka M, Borozan I, Brewer DS, Iskar M, Grundhoff A, Alawi M, et al. The landscape of viral associations in human cancers. Nature Genetics. 2020 10.1038/s41588-019-0558-9	HPV integration and impaired antiviral defense drive cervical, bladder, and head-and-neck carcinomas	3.12	[67] Preprint [88]

**Table 2:** Recap of selected datasets and computational tools generated as part of the PCAWG project.

Name of datasets and tools	Description	Accession link
PCAWG landing page	This is the recommended starting point for users wishing to access the PCAWG datasets *via* a single uniform web interface and a high-performance data download client. It provides browsing, download, and usage information for frozen PCAWG data files (Most of the data is open access with some controlled access requiring approval from the ICGC)	https://dcc.icgc.org/pcawg
Cancer Genome Collaboratory cloud portal	Cancer Collaboratory is an academic cloud-based access to the PCAWG dataset, excepting the TCGA-originated portion of the controlled data tier (see Bionimbus) (Open and controlled access)	https://cancercollaboratory.org/
The Bionimbus	Bionimbus is a cloud portal of protected data for cloud-based access to the TCGA-originated portion of the controlled data tier (Controlled access)	https://bionimbus-pdc.opensciencedatacloud.org
UCSC Xena data portal	UCSC Xena is a data portal for visualizations and analyses to integrate omics data generated by the PCAWG Consortium, including copy number, gene expression, gene fusion, promoter usage, simple somatic mutations, large somatic structural variation, mutational signatures, and phenotypic data	https://pcawg.xenahubs.net
Expression Atlas	Expression Atlas is an open science resource to find information about gene and protein expression. It enables queries across different tissues, cell types, developmental stages, and experimental conditions, across thousands of publicly available RNA-seq, microarray, and proteomics datasets	https://www.ebi.ac.uk/gxa/experiments?experimentSet=Pan-Cancer
PCAWG-Scout	PCAWG-Scout is a data portal that provides a framework to make on-demand, in-depth analyses over the open access PCAWG data	http://pcawgscout.bsc.es/
Chromothripsis Explorer	The Chromothripsis Explorer portal enables exploration of patterns of chromothripsis in the PCAWG dataset	http://compbio.med.harvard.edu/chromothripsis/
Cancer LncRNA Census	The Cancer LncRNA Census is an ongoing effort to identify and catalogue lncRNA genes which have been causally implicated in cancer	https://www.gold-lab.org/clc
PCAWG Core Pipelines	This Dockstore site contains binaries, source code, and documentation for the open source software tool for all core alignment, QC, and variation-calling pipelines used by PCAWG packaged as portable binaries using Docker and described using workflow description languages	https://dockstore.org/organizations/PCAWG/collections/PCAWG
Overture suite software tool	Overture comprises a set of open source tools for efficiently managing large genomic datasets and transferring them efficiently and reliably across the Internet	https://www.overture.bio/
Butler software tool	Butler is a workflow framework that facilitates large-scale genomic analyses on public and academic clouds while offering comprehensive error detection and self-healing capabilities (reviewed in Suppl. Text Chap. 2.1)	https://github.com/llevar/butlerl
SVclone software tool	SVclone is a computational method for inferring the cancer cell fraction of structural variant (SV) breakpoints from whole genome sequencing data	https://github.com/mcmero/SVclone
DriverPower software tool	DriverPower is a tool used to discover potential coding and non-coding cancer driver elements from tumor whole genome or whole exome somatic mutation sets	https://github.com/smshuai/DriverPower
TrackSig software tool	TrackSig is a computational framework to infer changes in somatic mutational signatures over time	https://github.com/morrislab/TrackSig
ActivePathways tool	ActivePathways is a tool for multivariate pathway enrichment analysis that identifies gene sets, such as pathways or Gene Ontology terms, prioritizes genes based on the significance of signals from the omics datasets, and performs pathway enrichment analysis of these prioritized genes	https://github.com/reimandlab/ActivePathways

All resources described are open accesses, unless otherwise stated in this table

## PCAWG cohort description and methodologies

The PCAWG collected WGS data with a mean read coverage of 39× from 2605 primary tumors and 173 metastatic lesions, as well as healthy tissue control. All the sequences obtained within the framework of the PCAWG Project belong to 38 different histological cancer subtypes. RNA-sequencing data were available for 1222 donors. The gender distribution of the final cohort is 55% male and 45% female donors, with a mean age of 56 years yet a wide demographic range from 1 to 90 years old individuals. More information on the cohort, storage, and handling of the data can be found in the Supplemental Text Chapter 1. To identify somatic mutations, computational pipelines were used across all 6835 samples to call: (1) somatic single-nucleotide variations (SNVs), (2) small insertions and deletions (indels), and (3) copy number alterations (CNAs) and structural variations (SVs). Other characterized features include somatic retrotransposition events, mitochondrial DNA mutations, and telomere length. RNA-sequencing data were uniformly processed to call transcriptomic alterations. Germline variants identified by the three separate pipelines included single-nucleotide polymorphisms, indels, SVs, and mobile-element insertions. More details on the pipelines are available on Pan-Cancer Analysis of Whole Genomes page (https://dcc.icgc.org/pcawg) and on ICGC DCC DOCS page ( http://docs.icgc.org/pcawg/data/) and can be found in Table 2, with description of each computational tool, methodology, and software generated detailed in the supplemental text. For a user guide to the visualization and exploration of the PCAWG dataset, please refer to ICGC DCC DOCS page (http://docs.icgc.org/pcawg/data/) and reference [1].

## Review of PCAWG findings in the major publications

The articles published on the 5th of February 2020 in *Nature* and partner journals as part of the PCAWG project report the most comprehensive evaluation of cancer mutations using WGS to date. The original articles published in the context of the PCAWG project can be accessed on the Nature website PCAWG landing page (www.nature.com/collections/afdejfafdb). Open-access pre-prints, which are non-peer-reviewed earlier versions of these publications, were deposited on bioRxiv and are widely accessible.

### Main paper: genetic changes in cancer by Pan-Cancer Analysis of Whole Genomes

“Pan-Cancer Analysis of Whole Genomes” is the key publication of the PCAWG project that mapped cancer-specific genetic changes across the WGS cohort teasing out driving mutations from passenger mutations that do not play an overt role in cancer [2]. To accomplish this task, a new software package, DriverPower, combines two state-of-the-art methods, mutational burden and functional impact evidence, to identify a cancer driver [3]. DriverPower’s innovative approach takes into consideration increased frequency over tumor background mutation rate as well as predicted impact on genomic functions [3]. This blended method addresses the issues of finding both recurrent and rare driving events, as well as those that occur in poorly understood or annotated regions. Nonetheless, all methods designed to date including DriverPower operate on the premises of neutral selection of genomic elements during model training. Instead, it is extremely likely that mutations reflect a balance between positive and negative selection, and accounting for a neutral selection and failure in correcting for cross-selective pressures reduce the overall sensitivity of existing methods. Furthermore, the assumption of functional relevance based on our still limited current knowledge should also be considered. DriverPower detailed review can be found in Supplemental Chap. 2.2, with more information available at the Github DriverPower page (https://github.com/smshuai/DriverPower) and in the original publication [3].

**Fig. 2:**
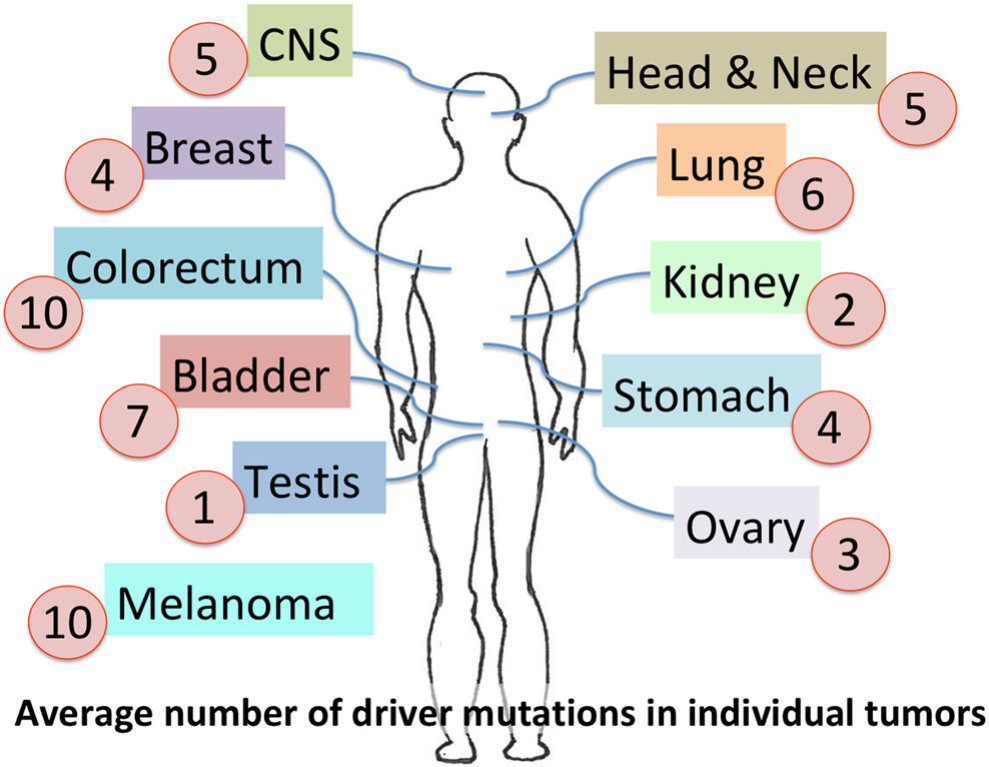
Number of mutations that drive each cancer. At least one driver mutation was found for 95% of all cancers, with an average of 4-5 driver mutations for each type of cancer [3]. Depicted are examples of cancers that have different amount of driver mutations promoting carcinogenesis. Depending on the type of cancer, anywhere from one to ten driver mutations are required for the tumor to develop.

Over 95% of tumors contained at least one and on average 4–5 identifiable driver mutations, indicating that generally no single cellular program directs cancer’s behavior. Instead, several changes impinging on multiple pathways enable each individual cancer to form [3]. Yet, each tumor differs considerably in the number of driver mutations needed to promote carcinogenesis (Fig. 2). The number of driver mutations is largely associated with the tissue’s proliferative status and surrounding micro-environment of the cell [4]. Intuitively, the number of drivers varies not only across tumors but also along the cancer timeline, with more advanced stages generally showing a higher burden of driving mutations [4]. The study found several common biological pathways involved in cancer: (1) chromothripsis, whose mutational signature of clustered structural variants seems to arise early in cancer; (2) telomere maintenance, with the majority of cancer mutations impinging on the TERT gene and its promoter region but also affecting the alternative lengthening of telomeres (ALT) pathway and other yet unidentified pathways to regulate telomere length in cancer [5]; (3) germline variants that genetically predispose individuals to an increased mutational burden, often due to malfunctioning DNA repair factors – with important preventive implications.

Germline variants were identified by three separate pipelines and included single-nucleotide polymorphisms (SNPs), indels, SVs, and mobile-element insertions [2]. 17% of patients had germline protein-truncating variants (PTVs), with biallelic inactivation happening in ~4% of these patiences due to further somatic alteration on top of a germline PTV affecting known cancer-predisposition genes (such as *BRCA1, BRCA2*, and *ATM*). The study also highlighted the complementarity between germline mutations in their influence on somatic mutation rate and pattern. Several examples were identified, including germline APOBEC3B-induced mutagenesis across all cancer types. *BRCA2* and *BRCA1* PTVs were associated with an increased burden of small somatic deletions, tandem duplications, and templated insertions in breast and ovarian cancers as well as other tumors like adenocarcinomas of the prostate and pancreas. *MBD4*, a DNA repair gene that deals with mismatches within methylated CpG sites, showed rare germline PTVs with an expected increased rate of somatic C to T mutations at the pan-cancer level. Finally, the PCAWG analysis uncovered 114 germline source L1 elements capable of active somatic retrotransposition, where some events caused deletion of the tumor suppressor gene *CDKN2A* [2]*.*

For tumor suppressor genes, including *P53* – the single highest mutated gene across all cancers – the Knudson two-hit hypothesis [6] has been further substantiated, where both alleles were found mutated or inactivated. Notably, most driver mutations were mapped within the coding genome, with only ~ 13% of tumor-specific driving changes present within non-coding DNA. This is especially surprising in light of the recently established genome regulation through SNPs in enhancers and ncRNAs, topologically associating domains (TADs), and spatial organization of non-coding loci that can affect gene expression [7]. Despite the depth and quality of the work, the PCAWG Consortium failed to identify any driver mutation in 5% of all cancers, raising the tantalizing possibility that these tumors may have a yet-unknown genetic etiology, potentially involving new molecular pathways or unmapped regions of the genome. The approach used to distinguish passenger from driver mutations ranked the observed mutations based on recurrence, estimated functional consequence, and expected pattern of drivers in that element [2]. The reliance on the basis of prior knowledge of cancer-causing pathways may have biased the identification of driver mutations within functionally annotated loci over unmapped regions or genes that lack functional characterization. Further and more unbiased exploration may fill in the 5% gap in finding at least one driving mutations for every tumor. A recent report using the PCAWG data found that passenger mutations may cumulatively have an impact as cancer drivers [8], thereby partially explaining the unmapped 5% (Chap. 4). While much more remains to be established, this seminal publication furthers our understanding of cancer genetic and provides an important and lasting resource for all cancer researchers, with profound implications to advancing diagnosis, treatment, and overall management of cancer.

### Hidden in the genome: non-coding driving mutations in cancer

For a long time, we wondered if we were missing a key side of the picture in cancer genetic. Cancer sequencing was limited to whole exome sequencing (WES), which is less than 2% of the genome, potentially leaving a large blind spot open for important cancer-related changes. It was expensive, and overly complicated, to sequence and analyze the remaining 98%, especially for large cohorts of tumor samples and corresponding healthy-matched tissues. Yet, the discovery of driver mutations in the non-coding *TERT* gene across many cancer types [9] raised the possibility that there may be numerous other non-coding driver mutations. Surprisingly, however, the PCAWG Project found that mutations in non-coding genome are relatively infrequent drivers of cancer. Only 13% of all tumor samples were found to have non-coding mutations involved in cancer formation – beyond those in the *TERT* promoter, which I will discuss separately in Sect. 3.7 [10]. Notably, even mutations in the regulatory sequences surrounding cancer genes are relatively rare, except those involving *TP53* gene 5′ untranslated region (UTR) or the *TERT* gene [11–13], suggesting that there may be a selection bias to directly target cancer genes. One non-coding driving mutation was found to occur every 100 tumors, compared to one or more protein-coding mutation found in every tumor sample. Interestingly, certain non-coding drivers found in previous studies were shown not to be *bona fide* cancer-causing mutations but the outcome of less stringent methodologies or by-products of hyper-mutation processes without a driving power in cancer formation [10]. For instance, the previously reported non-coding RNA *NEAT1* [14] may not be a *bona fide* driver but may get picked up as subjected to localized mutational processes. The unexpected fact that the vast majority of cancer drivers occur in coding regions with few driver mutations found outside protein-coding genes [10] is a very important piece of information to encourage rapid, inexpensive, and less complicated WES as a routine practice in cancer management, with WGS being readily available when no driver is found by exome sequencing or if clinically warranted.

### The genetic fingerprints of human cancer

Alexandrov et al. [15] and Li et al. [16] focused on understanding the patterns of genomic aberrations that are cancer specific and the molecular processes underlying these mutational signatures. A total of 97 patterns of mutations, some of which are new, were uncovered using non-negative matrix factorization (NMF) of context-specific mutation frequencies [15]. Mutations ranged from a single base pair variant to long stretches of DNA alterations, including loss or gain [15] as well as structural variations (SV), where large amount of DNA is rearranged, reorganized, and altered in sequence and in order. This analysis, a first of its kind, included complex mutational patterns associated with cancer [16]. In both publications [15, 16], the analyses are limited to somatic mutations, excluding germline mutations –which were looked at separately [2]. The starting point for the study was to sieve through the entire collection of almost 85 million of cancer-specific mutations derived from 4645 whole genomes and 19,184 exomes encompassing most cancer types. Alexandrov et al. [15] mapped the overall mutational catalogues for each individual cancer genome and assigned signatures due to (1) exogenous agents, for example, tobacco smoking or ultraviolet (UV) light; (2) endogenous sources, including replication and repair of DNA double-strand breaks (DSBs) through non-homologous end-joining – an error-prone pathway that re-ligates broken ends of DNA without filling in the gaps causing loss of genetic information; and (3) defective DNA repair mechanisms, such congenital as mutations in *BRCA1* or *BRCA2* that increase base substitutions and deletions [17]. In alignment with previous reports [18, 19], mutational rate increases with cellular and organismal age and depending on the tissue proliferative activity. These are events that occur in parallel to carcinogenesis and were taken into account to faithfully tease out cancer-associated signatures from background mutagenesis. Notably, about half of the signatures identified by this study remain of unknown causes. This implies that there are still major gaps in our knowledge of cancer-specific mutagenesis, including chemicals, environmental agents, as well as molecular pathways that can cause cancer.

In Li et al. [16], they exclusively focused on the mutational process of structural variations (SVs), during which rearrangements delete, amplify, or reorder genomic segments ranging from single genes to entire chromosomes. SVclone, a new computational method for inferring SVs in WGS data, also provided the clonality of balanced rearrangements and SVs. The Svclone package is shared on Github SVclone page (https://github.com/mcmero/SVclone) (Supplementary Chap. 2.3). Through a series of mutation-subgrouping steps, they found 16 signatures of SVs. Aligning the cancer genomes to a reference-build hs37d5 human genome, they called each breakpoint demarcating SVs. From the annotated SVs, they backtracked to infer the pathways responsible to generate these clusters, including replication- and recombination-based processes. Interestingly, and in agreement with previous literature [20, 21], late replicating regions displayed SVs in the form of deletions and ensuing inversions. On the other hand, early replicating regions are more prone to unbalanced translocations and tandem duplications indicating differential replication dynamics and repair options – for instance, homologous recombination only becoming available for repair after the sequence is replicated. One prominent SV was a template insertion within one locus resulting in tandem duplication. For instance, in liver cancer, template insertions enable activation of the telomerase gene *TERT*, while in ovarian cancer, tandem duplication acts to disrupt *CDK12*. Other papers produced by the PCAWG addressed complementary aspects of SVs, including inference of positive selection acting on recurrently rearranged regions of the genome [10], how structural variants affect the transcriptome [22] and chromosome topology [23], patterns of somatic retrotransposition [24], and distribution of chromothripsis across cancer types [25], as described below.

### Carbon-dating tumors: the evolutionary history of a cancer

One of the most remarkable and informative analysis of the PCAWG was undertaken by Gerstung et al. [26] to map the evolutionary lifetime of each cancer in the cohort. To reconstruct mutational signatures and build a timeline for the evolution of cells within a tumor, a new method was developed called TrackSig [27] and subsequently improved as TrackSeqFreq – based on allele frequencies [28]. TrackSig was able to determine the occurrence and recurrence of mutations, even in absence of any difference in mutational signature activity, thereby inferring an approximate order in which the somatic mutations accumulate over time (in depth description of TrackSig, in Supplementary Text 2.4). The software is available on Github TrackSig page (https://github.com/morrislab/TrackSig). The first mutagenic driver event often happened years or even decades before cancer diagnosis, opening up important avenues for early detection and possibly prediction of cancer growth. This temporal pattern was built using clonal variants, where early mutations are contained more widely in the sample while late mutations are present only in tumor subclones. Notably however, immune clearance can influence the survival of clones, while competition shapes the overall representation of subclonal populations within the tumor mass, and these represent potential caveats in building a reliable timeline. Reconstruction of a hierarchical timeline of mutations showed that the earliest mutations relate to a small subset of recurrent drivers, including *TP53* gene – often enabled by early loss of chromosome 17 petite arm, telomerase gene *TERT, CDKN2A*, and *KRAS*. The small number of initial changes in cancer evolution suggests an epistatic fitness landscape constraining those first steps in cancer evolution. After this initial selection, the set of mutations and affected genes broadens throughout cancer development. Late-stage cancers follow increasingly diverse paths driven by rarer and tissue-specific driver mutations, as well as more extensive alterations, implying acquired tolerance to mutagenic burden over time. Interestingly, pancreatic neuroendocrine tumors are outliers, showing entire losses of chromosomes 2, 6, 11, and 16 as early events indicating a separate mechanism of mutagenesis. Some mutational signatures span the entire timeline of cancer evolution, from early to late stages. An example of that is the distinctive pattern of DNA alterations caused by APOBEC mutagenesis. The APOBEC family of cytosine deaminases leads to cytosine substitution to uracil on single-stranded DNA, and subsequent abasic site, and break or substitution to thymine or guanine [29]. Dramatic mutagenic events like whole genome duplications (WGD) were also seen in several tumors. Interestingly, however, the timeline of their occurrence varies widely, with ovarian cancer showing a latency of up to 30 years after WGD, suggesting that this event may happen throughout the entire female reproductive lifecycle and becomes a cancer driver only in synergism with other changes. WGD in cervical cancers, on the other hand, has the shortest latency, about 2 years before diagnosis with higher *solo* driving potential. Altogether this study presents the first large-scale genome-wide reconstruction of evolutionary timelines, from early and pre-cancerous lesions to late-stage tumors. The finding of common genetic events occurring many years before tumor diagnosis opens up potential clinical avenues for prevention.

### Beyond DNA: RNA alterations associated with cancer

#### Genomic basis of RNA alterations in cancer

RNA can hold important information not only on the cellular proteinacious output but also on its transcriptome, including non-coding transcripts and regulatory RNAs, which have been found to play diverse roles in cancer [30]. The PCAWG Transcriptome Core Group harnessed the PCAWG/ICGC/TCGA cohort of whole genomes jointly with information on tumors transcriptomes. RNA sequencing information were not available for all cancers, largely due to different inclusion criteria, but the PCAWG Project still yielded 1188 donors that enabled a simultaneous characterization of RNA changes specific to cancer and their association to underlying DNA changes – thereby inferring mechanistic bases for transcriptional alterations in cancer [22]. Various forms of RNA disruptions had been previously described in cancer, including overexpression [31] and fusions [32]. The authors found that transcriptome changes in cancer were caused by somatic copy number alterations (SCNAs) as the major driver of variation in both total gene and allele-specific expressions, accounting for 17% of all gene expression variation, followed by somatic single-nucleotide variants (SNVs) found in gene flanking regions (1.8%) and finally germline variants in 1.3% of all tumors [22]. Using 28 mutational signatures derived by Alexandrov et al. [15] (Chap. 3.3) part of the PCAWG Project for context-specific mutation frequencies, they were able to draw associations between most RNA changes and the underlying DNA mutational signatures for each specific tumor. It’s interesting that over the total 649 associations of somatic SNVs with gene expression in *cis*, 68.4% involved associations with flanking non-coding regions of the gene instead of direct SNVs in exons or introns within the gene, suggesting these more “direct” changes may not be tolerated and that flanking non-coding regions have important regulatory roles exploited during carcinogenesis. Furthermore, somatic mutations led to ~1900 splicing alterations. Depending on position and orientation, Alu sequences could readily generate splice sites, causing exonization. The authors also found a new “bridged-fusion” mechanism where a third genomic location bridges two genes resulting in a fused RNA product. For instance, new *CTBP2-CTNNB1* fusion was found to drive RNA alteration in a gastric tumor sample. Notably, while 82% of all gene fusions identified could be matched with specific genomic rearrangements, the remaining may directly occur at the RNA level, as *trans*-splicing, discontinuous transcription, frameshifts, or read-through events. This study uncovered cancer-associated alterations that would have been undetectable via DNA-only approaches, underscoring the importance of integrating transcriptome with WGS analysis for cancer studies [22].

#### Impact of cancer-associated SVs in altered gene expression

SVs can directly alter gene expression through fusion, gene rearrangements, or copy number alternations, as described in 3.5.1. Zhang et al.’s [33] work addresses the impact of somatic SVs on gene expression in cancer looking exclusively at events where the breakpoint occurs outside of the gene yet drives changes in expression through modulation of regulatory elements. Using high-coverage whole-genome analysis of 1220 cancers for which transcriptomes were also available, the authors found pervasive misregulation of gene expression due to cancer-specific *cis*-regulatory alterations. SV breakpoints that are present within 100 kb of a gene were sufficient to impact its expression and regulation. Notably, the mechanism of *cis* regulatory disruption using SV-induced breakpoints led to an increase, rather than decrease, in gene expression. In addition to copy number amplification, SVs induced changes in the amount or spatial positioning of enhancers and other regulatory elements. Repressor elements were found to be directly inactivated by SV breakpoints. Accumulation of DNA methylation in the proximity of the promoter region due to SV was also found. Cancer-associated genes that were upregulated *via* these mechanisms include *TERT*, *CDK4*, *CD274*, *ERBB2*, *IGF2*, *MDM2*, and *PDCD1LG2.* The authors found melanoma, stomach, sarcoma liver biliary, and kidney cancers to rely on breakpoint for telomerase activation, largely through DNA methylation that juxtaposes the locus to strong enhancer elements. For *MDM2*, on the other hand, increased DNA methylation did not augment enhancer contacts, begging the questions as to the multiple ways in which DNA methylation – perhaps through large scale chromatin remodeling – leads to increased transcription. Altogether, the PCAWG repository represents a valuable resource to gain further insights into the global impact of non-exomic alterations with special attention to SVs as a widespread mechanism for enhancer hijacking and altered DNA methylation to drive cancer [33].

### Endogenous jumping elements’ role in cancer mutagenesis

Integration of retrotransposons has long been associated with cancer. Using the PCAWG cohort, 35% of all cancer samples showed evidence of having acquired at least one retrotransposition event, with a total of 19,166 somatically acquired retrotransposition causing diverse types of mutagenesis [24]. Long interspersed nuclear element (LINE-1) insertions emerged as the most frequent type of somatic SV in esophageal adenocarcinoma and the second most frequent in head-and-neck and colorectal cancers. Aberrant LINE-1 integrations can induce deletion of megabase-scale regions within a chromosome [34, 35], which can lead to the loss of tumor suppressor genes and can induce complex translocations and large-scale duplications. Somatic retrotranspositions can also initiate breakage-fusion-bridge cycles, leading to high-level oncogene amplification and possibly chromothripsis [36] (Chap. 3.11). LINE-1 elements capable of active somatic retrotransposition showed evidence of insertions as well as single-nucleotide polymorphisms in strong linkage disequilibrium. The mutagenic potential of these “hot” L1 elements was termed Strombolian and Plinian in analogy to patterns of volcanic activity, the former showing frequent small-to-modest eruptions in cancer samples while the latter, Plinian, with rare yet aggressive somatic activity. Each PCAWG donor bears between 5 and 7 elements with hot activity, but only 38% (1075 out of 2814) of PCAWG donors shows a Plinian element. The new evidence calls for a deeper understanding of population-based polymorphism in both Strombolian and Plinian elements, with WGS being a valuable option for at-risk population. Yet, we need to fully understand other synergistic features that, together with retrotransposable activity, can drive carcinogenesis. L1 retrotransposition can be a key factor in remodeling the cancer genome and should be taken into consideration through the development of human tumors [24].

### Key role for telomere maintenance mechanisms in cancer

One of the hallmarks of cancer is the ability to achieve replicative immortality and avoid telomere shortening with ensuing cellular senescence and proliferative arrest. This is accomplished through mutagenesis associated with telomerase, which is upregulated in ~85% of all human cancers by different genetic mechanisms [5]. The remaining 15% of cancers use a different way to maintain telomere sequences, called alternative lengthening of telomeres (ALT) pathway. ALT is a highly mutagenic process that promotes continuous non-allelic recombination to generate telomeric sequences of heterogeneous lengths and containing diverse telomere variant repeats (TVRs) [37]. Loss-of-function mutations in the chromatin remodeling genes *ATRX* (α-thalassemia/mental retardation syndrome X-linked) and *DAXX* (death domain-associated protein) [38] inhibit the ALT pathway, indicating their contribution to ALT. Using WGS data, the PCAWG study (1) determined telomere content, (2) searched for mutations associated with different telomere maintenance mechanisms, (3) systematically detected 2683 somatic telomere insertions, (4) found previously undescribed singleton TVRs, and (5) identified enrichment of TVRs through different telomere maintenance mechanisms [5]. Using a relatively simple method, the authors counted all the 100 bp reads containing at least six telomere repeats representing approximately half of the read (inevitably missing some reads which were only partially overlapping, likely positioned proximal to telomeric loci). Bait sequences used were the canonical telomere repeat TTAGGG and the three most common TVRs: TCAGGG, TGAGGG, and TTGGGG. They found that only 16% of tumors exhibited somatic mutations in at least one of *ATRX*, *DAXX*, and *TERT*. This striking finding implies that many players contributing to mechanisms of telomere’s maintenance, and especially those associated with cancer, are still unknown. Buried in the supplemental information of this publication [5] can be found the long-standing evidence of telomere attrition during organismal aging, with telomere content from healthy control sequences significantly anti-correlating with age. Because the cohort spans 1 to 90 years old individuals, the authors noted that the age effect is accounted for and bears little contribution to the strong correlation between the altered telomere content in the tumor versus control samples. Almost all tumor samples show a lower telomere content than the matched control regardless of age. The highest telomere content increase was seen in osteosarcomas and leiomyosarcomas, while particularly low telomere content was found in colorectal adenocarcinoma and medulloblastoma, indicating heterogeneity across tumors, which is likely derived by the telomere maintenance mechanism operating within the sample. Indeed, on average, ALT mutants gained telomere content, while telomere sequences were generally lost in telomerase cancers. Focusing on the samples with no clear underlying mechanism, there is a wide distribution of *TERT* expression. Some tumors show higher telomerase expression than *TERT* mutated samples but no obvious telomerase mutations, implying other ways to regulate telomerase that have not been uncovered yet. Further, a subset of cancers shows extremely low *TERT* expression, possibly suggesting they belong to ALT through mutations outside *ATRX* or *DAXX* or to completely unknown telomere maintenance pathways. Telomere insertions found in a third of all samples were mutagenic with 44% of interstitial telomeres associated with coding sequences including tumor suppressor genes and 8% of these directly disrupting exons. In addition to *ATRX* and *DAXX*, other genes were found co-mutated in association with telomere maintenance pathways [39–45] (details in Supplemental Table 1). The study also investigated TERRA, long non-coding telomeric repeat-containing RNA, which is elevated in ALT-positive tumors and aberrantly transcribed from telomere insertions [5, 46]. Finally, they looked at TVR across all tumor samples, including TGAGGG, TCAGGG, and TTGGGG, which are known to be enriched in proximal telomeric regions where the canonical TTAGGG starts to diverge. They also found novel aberrant TVRs with significantly higher counts in ALT samples. However, the majority of telomere insertions may be passenger mutations frequently located at copy number–neutral sites and found in late subclones. This work confirms regulation of telomeres as an important tumor-suppression mechanism, particularly in tissues with low steady-state cellular proliferation in which a clone must overcome this constraint to achieve replicative immortality.

### Mitochondrial DNA in cancer

Cancer requires huge amount of energy for its sustained proliferative capacity. Mitochondria are thus very important for cancer progression. Within the human nuclear genome, mutations vary across tissues. In mitochondrial DNA (mtDNA), the mutation burden is more homogenous, with cytosine to thymine being the most abundant signature which derives from powerful endogenous mutational processes intrinsic to mitochondria oxidative metabolism and unique mechanisms of DNA repair [47, 48]. In the context of aging, mutations in mitochondrial genomes, similarly to the nuclear genome, are increasing over time. Although not highlighted by the authors, the paper presents a dramatic spike in mutational burden in the mitochondrial genomes after 40 years of age, of unknown etiology [49]. While the cohort has less “under 40s” WGS overall, the age distribution [50] is unlikely to account for this striking, sudden, and synchronous appearance in mitochondrial mutations which remains unexplained. In each tissue and across different types of cancer, Yuan et al. calculated the number of mutations in the nuclear DNA and correlated this with that of the mitochondrial genome [49]. In addition to the unified C to T mutational signature, truncating mutations were found to be remarkably enriched in kidney, colorectal, and thyroid cancers and associated with the activation of critical signaling pathways. Interestingly, they also found different patterns of mtDNA mutations, for instance, between two different types of breast cancer, underscoring that tumors from the same tissue of origin could be genetically widely different in spite of having often similar, potentially unspecific, therapeutic interventions. Investigation into novel hypermutated cases revealed that fragmented mtDNA leaks into the cytoplasm and subsequently finds its way into the main nucleus in a chromothripsis-like event called NUMT (nuclear mitochondrial DNA sequences), which is seen in 2% of all cancers. Skin and lung cancers showed large number of integrations of mitochondrial DNA in the nuclear genome. Once the mitochondrial DNA integrates, they found that deletion, truncation, or other SVs were generated in the nuclear genome, including direct disruption of genes such as *ERBB2* – which is also a therapeutic target providing avenues for chemo-resistance. The authors also looked at RNA sequencing data from entire cohorts comparing co-expression pattern analysis to determine the highest changes correlated to mtDNA disruption by GO-term gene pathways. Perhaps unsurprisingly, oxidative phosphorylation, electron transport in mitochondrial membrane, DNA repair, and cell cycle genes were all upregulated in mitochondrial cancers [49]. To date, this study [49] represents the broadest mutational landscape of mitochondrial genomes whose contribution to carcinogenesis should not be underestimated.

### Architectural changes in cancer genomes

DNA inside the nucleus is spatially organized into topologically associating domains (TADs) that dictate a concerted pattern of expression and chromatin modifications, with cohesin and CTCF boundaries separating different domains [51]. While TAD disruptions in human cancers can lead to misregulation of gene expression, their frequency was found to be rather low, with only certain cancer subtypes showing SVs to span TADs [23]. This is surprising in spite of the great effort using WGS and our current understanding of genome regulation through chromatin folding. In the cases of TAD disruption, they found that SVs can lead to complex rearrangements that can result in the fusion of discrete TADs. Deletions were found to occur within the same TAD, while duplications tended to span regions across different TADs. These results suggest that mechanistic differences may underlie the generation of SVs, thereby associating different types of SVs with specific location within or outside the TADs. Hi-C data also showed that complex rearrangements lead to overall disruption of chromatin architecture, yet these events remain relatively rare across cancers. This may be due to the fact that only 14% of these boundary deletions resulted in a strong change in expression in nearby genes, making TAD disruption an inefficient way to carcinogenesis. Altogether, this study shows that TAD disruption happens in specific tumors and only subtly influences gene expression in the context of cancer.

### Common pathways and molecular networks affected in cancer

Next, the PCAWG Project analyzed the impact of mutations on cellular networks [52]. While non-coding cancer driver mutations are less well characterized and represent a minor proportion of the identified drivers, multi-faceted analyses across 2583 whole cancer genomes allowed to interpret pathway and network enrichment derived from these changes, using prior knowledge of genes and biological processes [53]. For this analysis, ActivePathways, a data fusion technique for multivariate analysis, was utilized for the discovery of the pathways across multiple datasets that are significantly enriched (more detailed description available in Supplemental Chap. 2.5). The ActivePathways software is available on Github’s ActivePathways page: https://github.com/reimandlab/ActivePathways [54]. Similar to what was found in the main PCAWG publication [3], the majority of cancer driver mutations are protein coding, with 79% of the cohort showing enrichments in pathways supported by protein coding genes [55]. Yet, this was integrated with non-coding mutations in genes that also contributed to uncover frequently mutated biological processes and pathways [52, 54–56]. The few recurrent non-coding mutations were most notably found within *TERT* promoter and 93 genes harboring non-coding mutations that cluster into several modules of interacting proteins. Noteworthy examples of the latter are promoter mutations associated with reduced mRNA expression in *TP53*, *TLE4*, and *TCF4*. This reinforces the notion that even non-coding mutations, which are seemingly passenger mutations, can collectively drive tumor formation [7]. Interestingly, key cancer-associated processes had variable proportions of coding and non-coding mutations, with chromatin remodeling and proliferation pathways altered primarily by coding mutations, while developmental pathways, including Wnt and Notch, altered by both coding and non-coding mutations. RNA splicing, on the other hand, was primarily altered by non-coding mutations. This indicates that different upstream mutational pathways are specific to selected cancer-driving pathways [56]. When non-coding driver mutations were analyzed separately for UTRs, promoters, or enhancers, over half showed enriched pathways. This suggests that pathway analysis is an attractive strategy for bringing to light the contribution of the non-coding regions of the cancer genome [57–60]. This analysis also highlighted immune response and anti-apoptotic signaling as potentially prognostic molecular pathways across breast cancers. Further integration of ChIP-seq and RNA-seq data derived from healthy tissues on the Hippo pathway identified processes related to stem cell regulation and tissue regeneration [61].

Collectively, this work revealed that many coding and non-coding mutations converge to affect or hijack selected pathways to drive carcinogenesis, with important therapeutic interventions harnessing synthetic lethality and synergism found between factors in specific tumors.

### Chromothripsis: a single initial catastrophic event to drive cancer

A key finding from the PCAWG Project was uncovering chromothripsis as a key driver of cancer. Chromothripsis is a mutational phenomenon characterized by fragmented DNA intergration in the genome after leaking from the main nucleus. This phenomenon results in massive, clustered genomic rearrangements that have been found in cancer and other diseases. Early evidence from low-resolution copy number data found chromothripsis as a rare event in cancer [62]. This was corroborated by the facts that chromothripsis was only recently discovered in spite of its dramatic mutational signature; furthermore, chromothripsis requires upstream cytotoxic events for loss of DNA that, during mitosis or in cases of nuclear envelop fragility, leaks out of the main nucleus to give rise to micronuclei (MNi), cytoplasmic entities containing nuclear DNA [63]. Failure to successfully replicate MNi results in fragmented DNA becoming available for reintegration into the main nucleus in the following cell cycle, an event that happens for about a third of all MNi. Thus, after a micronucleus is formed, chromothripsis has a high chance to occur. Chromothripsis was recently shown to be triggered by single mitotic errors such as the breakage of an anaphase chromosome bridge during cell division [36], likely a relatively common occurrence in cycling cells and a widespread one in cancer. Indeed, as part of the PCAWG, chromothripsis was found to be incredibly pervasive across cancers, with a frequency of more than 50% in some cancer types. Patterns across 2658 tumors from 38 cancer types using WGS data show that chromothripsis profiles display oscillations between localized structural alterations in a high proportion of events [25]. Mutational signatures of chromothripsis are mainly associated with non-homologous end joining (NHEJ) that works by enabling reintegration of DNA fragments by ligating them back into the nuclear genome. Additional signatures associated with chromothripsis are replication-associated processes and templated insertions, suggesting other mechanistic modalities for mutagenic reintegration of DNA into the genome, probably dictated by different cell cycle stages [64]. Chromothripsis induces direct disruptions of genes, including inactivation of mismatch repair–related genes, therefore setting in motion additional mutagenesis [25]. Thus, chromothripsis likely represents a relatively early event in tumor formation and a major driver of genome evolution in human cancer. Evidence of chromothripsis through identification of more complex structural alterations was found to contribute to oncogene amplification and translocations [65]. ShatterSeek allowed to identify chromothripsis events from WGS data by initial detection of intrachromosomal SVs to find clusters of interleaved rearrangements and then by taking both input copy number (CN) and SV calls. These profiles can be visually inspected for each candidate chromothriptic region found. All the chromothripsis calls reported can be visualized on the Chromothripsis Explorer page (http://compbio.med.harvard.edu/chromothripsis/), while the original code can be accessed on Github ShatterSeek page (https://github.com/parklab/ShatterSeek). This finding furthers our understanding of chromothripsis, both its etiology through different cytotoxic mechanisms and its consequences upon reintegration in the main nucleus, with profound implications in further understanding the temporal profile of cancer mutagenesis.

### Viral integration as a driver for cancer

Viruses have long been associated with cancer. This historic association has been at times misunderstood with theories inferring that all cancers were due to a virus [66]. The PCAWG project and uncountable earlier evidences have refuted this hypothesis, estimating less than 10% of all cancers attributable to viruses. Yet, viruses remain important drivers of a specific subset of human tumors, and viral integration with subsequent disruption of genes remains of high prevalence in cancer overall, with epidemiological studies suggesting that recurrent viral infections can be a major risk factor for certain cancers. Zapatka et al. systematically investigated potential viral pathogens using the integration of three independent computational pipelines to call for viral signatures associated with cancer [67]. Viruses were detected in 382 genome and 68 transcriptome datasets, a relatively small proportion of all two thousand five hundred cancer and over one thousand RNA samples [67]. High prevalence of known tumor-associated viruses such as Epstein-Barr virus (EBV) [68], hepatitis B virus (HBV) [69], and human papilloma virus (HPV; e.g., HPV16 or HPV18) [70] was confirmed. Interestingly, the author found that an impaired anti-viral defense may synergize with viral infection to drive carcinogenesis. HPV was found associated with APOBEC mutational signatures in cervical, bladder, and head-and-neck carcinoma. For HBV, HPV16, HPV18, and adeno-associated virus-2 (AAV2), viral integration was associated with local variations in genomic copy numbers, driving cancer by local gene amplification. Viral integration at the *TERT* promoter hijacks telomerase and increases its expression level. Finally, they found high levels of endogenous retrovirus (ERV1) expression being linked to a worse survival outcome in patients with kidney cancer, suggesting that viral infection initiates a cascade of additional genetic changes or is preceded by a relative weakness in the immune system that enables cancer to form and/or fosters its resistance to therapeutics [67]. Understanding the consequences, the synergizing features, and exact timeline between viral infection and cancer development is an extremely important piece of the puzzle that needs to be unveiled in order to prevent, and better handle, the clinical management of infections by oncolytic viruses [71].

### Non-coding RNA census in Pan-Cancer tumorigenesis

The ICGC/TCGA PCAWG Consortium focused on the identification of a specific type of non-coding RNAs, the long non-coding RNAs (lncRNAs), which have been shown to be dysregulated or mutated in tumor-specific manner with widespread effects on gene expression [72]. The PCAWG Project has identified a large number of unique cancer-associated lncRNAs [73]. A full, updated list can be accessed at the Cancer LncRNA Census (CLC) (https://www.gold-lab.org/clc), detailing lncRNAs that have validated roles as cancer drivers. This thorough compilation shows 122 GENCODE-annotated lncRNA genes that have well-established roles in cancer especially affacting gene expression. Of these, 77 were found to hold oncogenic potentials, while 35 had a tumor suppressive function. Interestingly, 10 lncRNAs exhibited both activities. Across all human cancers, the most widely observed lncRNAs were (1) HOX antisense intergenic RNA (HOTAIR), a lncRNA encoded in the *HOXC* gene that interacts with Polycomb Repressive Complex 2 (PRC2), a histone methyltransferase, for methylating and silencing various tumor suppressor genes; (2) MALAT1 (metastasis associated lung adenocarcinoma transcript 1) also known as NEAT2 (noncoding nuclear-enriched abundant transcript 2), a large lncRNA involved in alternative splicing, underscoring the widespread role of splicing alterations in tumorigenesis; (3) Maternally expressed gene 3 (MEG3) non-coding RNA that regulates cell proliferation through p53-dependent and p53-independent pathways, working as a putative tumor suppressor; and (4) oncogenic H19 (H19 Imprinted Maternally Expressed Transcript) is an evolutionarily conserved RNA gene affiliated with the lncRNA class which is found to induce cell survival pathways under specific stress conditions (reviewed in [74]). Altogether, the Cancer LncRNA Census by the PCAWG provides a comprehensive catalog and annotation dataof the lncRNAs found to hold a driving functional role in cancer development.

## The aftermath: additional work leveraging the PCAWG datasets

The work produced by the PCAWG Project represents a key resource for the scientific community, which is set to drive discoveries and to advance our understanding of cancer genetics for years to come. A testimony of this is new papers that have harnessed the access to the PCAWG sequences. Work published in Cell [8] just a few weeks after the publication of the PCAWG studies (it is however noteworthy that all PCAWG preprints hereby reviewed have been available and accessible online for one year or more ahead of publications [75–88]) harnessed the power of the large cohort to look at special types of mutations positioned in between clear driving mutations, with a functional role in cancer, and passenger mutations that do not contribute to carcinogenesis. They put forward the tantalizing hypothesis that a concerted collection of seemingly passenger, low functional impact alterations may collectively contribute to drive cancer. They found that the aggregated effect of passenger mutations plays a role in tumorigenesis beyond standard drivers. This finding also implies that the dichotomy passenger-driver when binning mutations may be unsatisfactory or may bury additional complexities associated with cancer-specific changes. They proposed that our current model of “drivers” and “passengers,” with only a few mutations in a tumor strongly affecting its progression while the remaining ones are inconsequential, may be misleading. While high- and low-impact variants clearly exist and can be determined, this new study highlights a third group of medium-impact putative passengers. Because the molecular impact on mutations correlates with the mutational signature and subclonal architecture, with early mutations being more likely to have a cumulative impact than later ones, those factors must be taken into consideration in concert. Through adapting an additive effects model from previous complex trait analyses, the aggregated effect of putative passengers, including undetected weak drivers, provides significant additional driving changes in cancer compared to the ones identified from the PCAWG initial analysis. Notably, this integrative framework found potential weak-driver mutations in the 5% of PCAWG samples lacking any well-characterized driver alterations, hyghlighting both the possibilities and complexities in fully deciphering the genetic changes that contribute to each cancer [3].

## Conclusions

By combining sequencing of the whole genomes with a suite of analysis tools (also see Supplemental Text), cancer’s genetic changes were comprehensively mapped by the PCAWG Consortium. The PCAWG Project identified the patterns of mutations that drive cancer, from single base pair changes to whole chromosomal rearrangements; the order in which they emerge in the lifetime of a cancer; the processes that have generated those mutations; and biological pathways altered to enable cancer formation, establishment, and progression. Using the knowledge of those mutations, which occur years, or even decades, before the tumor appears opens a window of opportunity for early cancer detection. Some countries are moving toward WGS of every cancer patient to guide treatment, yet this work also points to exome sequencing as a suitable initial analysis of cancer genetic identity, possibly complemented by RNA sequencing. The findings of the PCAWG Project represent a big step toward cataloguing all the major cancer-causing mutations with important implications for the future of precision cancer care. It also serves as a key resource for many other Pan-Cancer studies that have emerged analyzing the data from the original PCAWG cohort. And yet, the fact that drivers in 5% of cancers continue to remain mysterious comes as a reminder that there’s still more work to do. The recent paper addressing this point [8] that leveraged the PCAWG data is a prime example of how this repertoire will propel cancer research forward and is bound to generate more answers and questions.

The challenging next steps include connecting the cancer genome data to treatments and building meaningful predictors for patient outcomes. Much more data, potentially in tens of thousands of patients per tumor type, are needed to fully understand each cancer type – this is why shared data and resources (Table 2), like the PCAWG project, are essential stepping-stones. There is an urgency for international unified guidelines on medical, patients, and data handling that will facilitate this kind of work in the future [89]. The PCAWG adds promise to precision and personalized medicine, with the implementation of targeted therapies matched to the molecular and genomic profile of individual patients’ tumors. Therapeutic targeting can draw directly from the PCAWG key findings. First, the concept that tumor clustering is primarily based on cell of origin can help design therapeutic strategies based on molecular profiling of cancers instead of just relying on anatomical location. Genomics can also better define tumor clonal evolution and heterogeneity, and the information can be better integrated into therapy. Information on the immunogenicity of a tumor can also readily provide options for broad-spectrum treatments including immunotherapy and synergistic approaches. Liquid biopsies can harness the power of WGS to open new windows for earlier and more targeted interventions in cancer treatment but also opportunities for preventive screening. Similarly, comprehensive work like the one done in the context of the PCAWG Project unlocks the option of patient stratification based on genomic and molecular profiling to better select candidates for different trials of novel drugs or small molecule inhibitors, thus providing a better understanding of the response, resistance, and prognostic values of specific mutations and biomarkers. Although these therapeutic avenues are still largely speculative at this point, there is a sense of hope that these findings and others will ultimately vanquish, or at least rein in, *The Emperor of All Maladies* [90].

## Supplementary Information

ESM 1 (DOCX 33.7 kb)
