# Supplementary material for: Decoding human cancer with whole genome sequencing: a review of PCAWG Project studies published in February 2020
Source: Cancer Metastasis Rev. 2021 Jun 7;40(3):909–24. doi: 10.1007/s10555-021-09969-z (PMC8180541; doi:10.1007/s10555-021-09969-z)
Supplement: Supplementary file 1 — (DOCX 33.7 kb) [file 10555_2021_9969_MOESM1_ESM.docx]

**Supplemental material.**

**Decoding human cancer with whole genome sequencing**

***A Review of PCAWG Project studies published in February 2020***

Simona Giunta ^1,2^

**Correspondence to**: [simona.giunta@uniroma1.it](mailto:simona.giunta@uniroma1.it)

**Affiliations:**

1. Laboratory of Genome Evolution, Department of Biology & Biotechnology “Charles Darwin”; University of Rome Sapienza, Italy.
2. The Rockefeller University, 1230 York Avenue. New York, NY USA

Content:

**Supplemental text**

**Links and Resources**

**Supplemental Table 1**

**Supplemental Text**

**Supplemental Text 1. Review of PCAWG Computational Tools.**

***1.1 Open access data.***

The ICGC/TCGA PCAWG represents the largest, most comprehensive analysis of whole cancer genomes to date, involving non-coding regions, genes, site for non-coding RNAs and large-scale structural rearrangements. The project combined sequencing of over two thousand six hundred whole cancer genomes across 38 different types of tumors. Open-tier data can be viewed on PCAWG - ICGC Data Portal (<https://dcc.icgc.org/pcawg>) with instructions for obtaining access to the controlled-tier PCAWG data available in the DCC PCAWG documentation pages (http://docs.icgc.org/pcawg/data/). There is also an email ([dcc-support@icgc.org](mailto:dcc-support@icgc.org)) to inquire about data access. A very recent publication also provides a deeper description of principal tools developed and used in the course of PCAWG, called PCAWG-12 (1).

***1.2 Cohort description and data analysis.***

The PCAWG collected genome data from 2,834 donors but excluded 251 after quality assurance due to possible cross-contamination of the healthy tissue sample with cancer cells. Across donors, whole-genome sequencing data with a mean read coverage was 39× were available from 2,605 primary tumors and 173 metastatic lesions. 2,583 donors exhibited good quality data and were labeled as white-listed donors (2). RNA-sequencing data, on the other hand, were only available for 1,222 donors, obtained with a health-matched WGS sample for direct comparison for a total of 2,444 transcriptome samples (22). The gender distribution of the final cohort was the following: 55% male and 45% female donors, with a mean age of 56 years yet a wide demographic range from 1 to 90 years old individuals. To identify mutations, 3 established pipelines were used across all 6,835 samples to call: 1) somatic single- nucleotide variations (SNVs), 2) small insertions and deletions (indels), 3) copy-number alterations (CNAs) and structural variants (SVs). Other features isolated included 4) somatic retrotransposition events (24), 5) mitochondrial DNA mutations (49) and 6) telomere length (5). RNA-sequencing data were uniformly processed to call 7) transcriptomic alterations (22). 8) Germline variants identified single-nucleotide polymorphisms, indels, SVs. Pipelines can be accessed on PCAWG - ICGC Data Portal (<https://dcc.icgc.org/pcawg>) and specific computational tools are described below.

***1.3 Data portals and cloud-based resources***

The challenges in working with an intercontinental consortium pooling data from a collection of ~ 2,600 whole cancer genome sequences coming from 33 different countries over a decade (10) included 1) the size of the data, 2) rapid and effective sharing and 3) coordinating availability of both data and computational tools generated and improved as part of the project. These challenges were addressed using cloud computing. The data was stored in 13 data centers on three continents. The Sanger Institute, the main repository for the Project data, holds a usable storage of 65 petabytes (Pb), amounting to around 45 thousand computers. The mole of the data generated by the PCAWG is equally impressive to grasp. With a human genome sequenced every ~ 3 minutes, the raw genomic data produced around the world as part of the ICGC amassed more than 800 terabytes of genomic data, reaching two Pb in just five years. The Pan-Cancer study access the data and analysis tools on the cloud, using docker technology, packaging their software into Docker’s containers and porting them to run where the data was stored – in 13 data centers. These data centers included a mixture of commercial clouds, infrastructure-as-a-service, academic cloud compute, and traditional academic high-performance computer clusters. This set up enabled researchers across the participating data centres to uniformly align and perform variant calling on the ~ 5,800 whole genomes (2). Core pipelines were provided as reproducible, stand-alone packages, which have now been made available for download to the scientific community. In addition to data repositories for raw and derived datasets, portals for data visualization and exploration were generated to facilitate readily interpretation of the finding, now published (http://docs.icgc.org/pcawg/data/). Making such a large amount of data available to researchers across different jurisdictions, with diverse privacy, patients’ data handling and information legislations represented a challenge on its own. A code of conduct was specifically drafted for this project, with compliance from participating scientists, partners and associates; yet, this highlights a pressing need for international unified guidelines on medical, patients, and data handling, that will facilitate this work in the future (86). The ethical questions that emerged from could-computing and coordinated access to large patients cohorts like this one are beyond the scope of this review, yet it remains a profound and important aspect to reflect on for such projects.

**2. Computational tools**

***2.1 “Butler” for rapid cloud-based analysis of thousands genomes.***

Butler is a platform that facilitated the large-scale genomic analyses of datasets held in cloud-based workflows on Github Butler Page (https://github.com/llevar/butler). The **PCAWG** used Butler to run cancer genomics workflows simultaneously on all high-coverage whole genome samples collected by the pan-cancer project, worth ~ 725 TB of data, on the free, cloud-computing platform Openstack. Butler is an ingenious framework to run scientific workflow through clouds, enabling remote connection and coordinated international efforts to address the heterogeneous nature of the biological samples. Frequent artefacts generated upon in-depth analyses of sequences of heterogeneous quality, produced by different centers with varying operating procedures can cause significant failures of computational processes, limit unified and meaningful progress, affect the data processing rate and increase project duration and cost. A few noteworthy issues that have been reported by the PCAWG were 1) sequencing library artefacts, 2) sample contamination – where the healthy tissue may have been contaminated with cancer cells; and 3) non-uniform sequencing coverage that have resulted in data and software anomalies that challenged the pre-existing workflows. Butler served to provide an operational management toolkit that quickly identifies and resolves failures that are expected given dataset heterogeneity, as well as other unexpected caveats in dataset analysis using innovative anomaly detection and self-healing functions. Butler enabled access and processing of an extremely large cancer genome dataset in a time-efficient and uniform manner improving the efficiency of data processing and analysis by 43% compared to prior approaches. Code for Butler can be accessed on Github (https://github.com/llevar/butler) (Table 2).

***2.2 DriverPower – Refers to Chapter 3.1***

DriverPower (3) represents an innovative software package that uses mutational burden and functional impact evidence to identify a driver. Github DriverPower Page on <https://github.com/smshuai/DriverPower>. The key feature is the functional impact score to further increase the accuracy of driver discovery based on prior information of the loci functionality (3). However, this may also represent a substantial limitation in identifying driver mutations that are outside of previously known regions or regions for which, based on current knowledge, we can anticipate the functional relevance. This is especially true for mutations that are less penetrant or prevalent in the tumor population that would be harder to call. Nonetheless, using as many as 1373 genomic features derived from public sources, DriverPower's background mutation model is actually able to explain up to 93% of the regional variance in the mutation rate across multiple tumor types and notably, identify novel coding and non-coding drivers of cancers (2). The key improvement compared to previous methods is, again, a combinatorial approach that fuses two mutation significance testing methods. Instead of detecting signals of positive selection either through mutational burden, that would omit rare drivers, or functional impact tests, that would fail to map drivers in poorly annotated regions for which information is lacking, DriverPower used two algorithms for modelling the background mutation rate based on genomic features. The first algorithm was randomised lasso followed by binomial generalised linear model (GLM). The other algorithm was the gradient boosting machine (GBM), a non-linear and non-parametric tree ensemble algorithm, which outperformed GLM in tumors that have low level of mutations. Applying this method to the aggregated WGS data from 2658 cancers across 38 tumor types generated by the ICGC and TCGA projects, DriverPower outperformed six other published methods for both coding and non-coding driver discovery, and allowed to uncover 217 coding and 95 non-coding driver candidates, including novel drivers. Prior to analysis, the WGS data were re-analysed with standardised, high-accuracy pipelines to align to the human genome (reference build hs37d5). One limitation may be the reliance on correct and complete annotation of the reference genome, namely hs37d5 or hg19. Using a reference itself is problematic because it fails to encapsulate the intrinsic population heterogeneity. Importantly, to date, all methods operate on the premises of neutral selection of genomic elements during model training. Instead, it is extremely likely that mutations reflect a balance between positive and negative selection, and accounting for a neutral selection and failure in correcting for cross-selective pressures reduce the overall sensitivity of existing methods. Yet, DriverPower represents a robust and innovative framework for driver discovery, with special significance to the analysis of cancer genomes. Notably, DriverPower was used for major publications from the Project (10).

***2.3 SVClone – Refers to Chapter 3.2***

SVclone is a new computational method for inferring the cancer cell fraction of tumour structural variation from whole-genome sequencing data, the package is found on Github SVclone Page (<https://github.com/mcmero/SVclone>). Using this package, they explored the clonality of balanced rearrangements and structural variations (SV). This package was used to cluster SV of similar CCF by processing through five steps, as described here. BAM level information is used to 1) annotate to infer directionality of each breakpoint and classifying SVs using a rule-based approach; and 2) count the variant and non-variant reads from breakpoint locations. 3) Filtering removes SVs, or adds SNVs, based on prefixed parameters; 4) cluster of variants and 5) post-assign steps assigned SVs to the derived model. This pipeline allows to accurately determine the variant allele frequencies of both SV break ends, and simultaneously estimates the cancer cell fraction and SV copy number. Altogether, SVclone enables improved characterization of SV intra-tumor heterogeneity and may hold a prognostic value for specific cancers.

***2.4 TrackSig – Refers to Chapter 3.4***

To reconstruct mutational signatures and build a timeline for the evolution of cells within a tumor, a new method was developed called TrackSig (27). TrackSig was able to identify mutation populations across time from single bulk tumor sample and to determine the different frequencies even in absence of any difference in mutational signature activity. Reconstructing the evolutionary trajectories of mutational signature activities from WGS data from a single tumor sample using CCF corrected by copy number to infer an approximate order in which the somatic mutations accumulate over time. The software is available on Github TrackSig Page: (https://github.com/morrislab/TrackSig). Disentangling a temporal timeline for the occurrence of 1) driver events precede carcinogenesis, 2) driver mutations that confer a selective advantage, from 3) mutations that continuously accumulate in the genomes of our somatic cells, and also 4) cancer-specific passenger exacerbated by mutagenic events typical of cancers, is extremely complex from single, bulk tumor genome data. The new method, TrackSig, can achieve accurate reconstructions by taking into account properties and types of each mutation without clustering of mutation variant to reconstruct subclonal lineages. External or intrinsic mutational burdens to the cell can generate distinct mutational patterns, and this information can be harnessed to infer the evolutionary timeline along which clonal and then subclonal populations have moved toward cancer establishment. TrackSig is able to detect changes in signature activities across time using topic modeling and optimal segmentation. It does so by integrating a probabilistic inference of the mutation distribution and mutational activity ensuing specific mutations through known mutagenic processes. The method allowed classification into 96 different types of substitution and the trinucleotide context surrounding the mutated site. This ingenious approach reconstructs subclone architecture by back tracking from the mutational pattern, to the overall signature, all the way to mutagenic processes contributing to the generation of those signature activities. Notably, unlike prior methods, TrackSig groups mutations by their inferred prevalence in the cell population and partitions this timeline into clustering similar signatures. These features alleviate the dependency on optimal and homogenous sequencing depth, measurement noise and approximations derided by using bulk data from a single sample, thus outcompeting previous methods at estimating activities and identifying subclonal populations in complex scenarios such as branching evolution or violation of the infinite sites assumption. An evolution of the method, TrackSigFreq, was recently reported that identifies subclones using both mutation types and variant alleles frequencies (VAF) in (28). TrackSigFreq (28) extends TrackSig to allow it to detect change points between distinct subpopulations with little to no change in signature activity by incorporating information about mutation VAF density that would otherwise be missed. Collectively, these novel computational approaches that incorporate neutrally evolving mutations allow to map mutational signature activities over time and reconstruct evolutionary trajectories can ultimately be harnessed to predict cancer development, inform treatment choice and better understand disease progression.

***2.5 ActivePathways – Refers to Chapter 3.10***

High-throughput (omics) data analysis of The ICGC/TCGA PCAWG Consortium WGS data from 2658 cancers across 38 tumor types was performed to interpret pathway enrichment analysis using current knowledge of genes and biological processes. ActivePathways method, which uses data fusion technique for multivariate analysis, allowed the discovery of the pathways across multiple datasets that are significantly enriched. Pathway enrichment analysis identifies gene sets, such as pathways or Gene Ontology terms that are over-represented in a list of genes of interest. ActivePathways uses a data fusion method to combine multiple omics datasets, prioritizes genes based on the significance of signals from the omics datasets, and performs pathway enrichment analysis of these prioritized genes. The package is available on Github ActivePathways Page <https://github.com/reimandlab/ActivePathways> (52, 54). Using this strategy, pathways and genes supported by single or multiple omics datasets, as well as additional genes and pathways that are only apparent through data integration are detected. The analysis relies on statistical data fusion, rationalizes contributing evidence and highlights associated genes integrated genes with coding and non-coding mutations and revealed frequently mutated pathways and additional cancer genes with infrequent mutations. Integration of trascriptomics data over genomic mutations was done specially for breast cancers. Importantly, this analysis highlighted immune response and anti-apoptotic signaling as potentially prognostic molecular pathways in across breast cancers. Further integration of ChIP-seq and RNA-seq data derived from healthy tissues on the Hippo pathway identified processes related to stem cell regulation and tissue regeneration (61). ActivePathways approach operates by considering the following specific input: 1) a matrix of gene P-values for different omics datasets, and 2) a collection of gene sets corresponding to biological pathways and processes. It identified at least one significantly enriched process or pathway in ~ 89% of samples through an integrative priority list with many shared genes connected into broader biological networks. Similar to what was found in WGS analysis where the majority of cancer driver genes have frequent protein-coding mutations, 79% of cohorts showed enrichments in pathways supported by protein coding genes (52). In summary, ActivePathways represents a versatile and integrative method for pathways analysis that improves systems-level understanding of cellular organization in health and disease through integration of multiple molecular datasets and pathway annotations (52).

**Links and Resources:**

- Pan-Cancer Analysis of Whole Genomes landing page from Nature collection. Available online: <https://www.nature.com/collections/pcawg/>
- Pan-Cancer Analysis of Whole Genomes datasets and resources from Nature collection. Available online: <https://www.nature.com/collections/afdejfafdb/datasets-and-resources>
- ICGC portal from International Cancer Genome Consortium. Available online: <https://icgc.org/>
- TCGA portal from The Cancer Genome Atlas. Available online: <https://www.cancer.gov/about-nci/organization/ccg/research/structural-genomics/tcga>
- Expression Atlas portal from The European Bioinformatics Institute. Available online: [www.ebi.ac.uk/gxa/home](https://www.ebi.ac.uk/gxa/home)
- PCAWG Data Portal from Pan-Cancer Analysis of Whole Genomes. Available online: [dcc.icgc.org/pcawg](https://dcc.icgc.org/pcawg)
- PCAWG-Scout portal from Pan-Cancer Analysis of Whole Genomes. Available online: [pcawgscout.bsc.es](http://pcawgscout.bsc.es/)
- PCAWG Xena Hub page from The University of California, Santa Cruz. Available online: [pcawg.xenahubs.net](https://xenabrowser.net/datapages/?hub=https://pcawg.xenahubs.net:443)
- Chromothripsis Explorer Page from Department of Biomedical Informatics Harvard Medical School. Available online: <http://compbio.med.harvard.edu/chromothripsis/>
- ShatterSeek Page from Github. Available online: <https://github.com/parklab/ShatterSeek>
- [Pan-Cancer Analysis of Whole Genomes](https://dockstore.org/organizations/PCAWG) / Tools and Workflows from [Pan-Cancer Analysis of Whole Genomes](https://dockstore.org/organizations/PCAWG). Available online: https://dockstore.org/ organizations/PCAWG/collections/PCAWG.
- Overture software package from The Ontario Institute for Cancer Research. Available online: https://www.overture.bio/
- ICGC DCC Docs page from Pan-Cancer Analysis of Whole Genomes. Available online: <http://docs.icgc.org/pcawg/data/>
- European Genome Phenome Archive from The European Bioinformatics Institute : https://www.ebi.ac.uk/ega/search/site/pcawg (under accession number EGAS00001001692).
- Cancer Genome Collaboratory from The Ontario Institute for Cancer Research. Available online: <https://cancercollaboratory.org/>
- Bionimbus Protected Data Cloud from The University of Chicago. Available online: <https://bionimbus-pdc.opensciencedatacloud.org>
- Expression Atlas Experiment Page from The European Bioinformatics Institute. Available online: <https://www.ebi.ac.uk/gxa/experiments?experimentSet=Pan-Cancer>
- Cancer LncRNA Census Page from Gold Laboratory-Universitätsspital Bern.. Available online: <https://www.gold-lab.org/clc>
- SVclone Page from Github. Available online: <https://github.com/mcmero/SVclone>
- DriverPower Page from Github. Available online: <https://github.com/smshuai/DriverPower>

**Supplemental Table**

| Gene | Association With Telomere Maintenance Pathway | Reference |
| --- | --- | --- |
| *TP53* | p53 was also found to inhibit telomerase, thus truncated p53 was associated with high levels of TERT3 | Guièze R, Pages M, Véronèse L, Combes P, Lemal R, Gay-bellile M, et al. Telomere status in chronic lymphocytic leukemia with TP53 disruption. Oncotarget. 2016. |
| *ATRX/DAXX* | The death domain-associated protein DAXX and the chromatin remodeling factor ATRX are specifically associated with the H3.3 deposition machinery. The loss of function of ATRX and/or DAXX correlates with the ALT phenotype | Lovejoy CA, Li W, Reisenweber S, Thongthip S, Bruno J, de Lange T, et al. Loss of ATRX, genome instability, and an altered DNA damage response are hallmarks of the alternative lengthening of Telomeres pathway. PLoS Genetics. 2012. |
| *PLCB2* | The yeast homologue of PLCB2, PLC1, was found in a TLC1 knock out study | Hu Y, Tang HB, Liu NN, Tong XJ, Dang W, Duan YM, et al. Telomerase-Null Survivor Screening Identifies Novel Telomere Recombination Regulators. PLoS Genetics. 2013. |
| *MEN1* | The tumor suppressor menin, encoded by MEN1, was reported to negatively regulate telomerase by binding to the TERT promoter | Ramlee MK, Wang J, Toh WX, Li S. Transcription regulation of the human telomerase reverse transcriptase (hTERT) gene. Genes. 2016. |
| *TSSC4* | TSSC4 was found in close proximity to RAP1 by a PCA/BiFC assay | Askree SH, Yehuda T, Smolikov S, Gurevich R, Hawk J, Coker C, et al. A genome-wide screen for Saccharomyces cerevisiae deletion mutants that affect telomere length. Proceedings of the National Academy of Sciences of the United States of America. 2004. |
| *RB1* | Both telomeric chromatin compaction and telomeric repeat-containing containing RNA (TERRA) transcription are dependent on RB1 expression | Gonzalez-Vasconcellos I, Schneider R, Anastasov N, Alonso-Rodriguez S, Sanli-Bonazzi B, Fernández JL, et al. The Rb1 tumour suppressor gene modifies telomeric chromatin architecture by regulating TERRA expression. Scientific Reports. 2017. |
| *ABCC8* | The yeast homologue of ABCC8, YOR1, was found in two independent deletions screens in S. cerevisiae | Katzmann DL, Hallstrom TC, Voet M, Wysock W, Golin J, Volckaert G, Moye-Rowley WS. Expression of an ATP-binding cassette transporter-enconding gene (YOR1) is required for oligomycin resistance in Saccharomyces cerevisiae. Molecular Cell Biology. 1995. |

**Supplemental table 1. Cancer-specific genes associated with telomere maintenance pathways.** Most had been previously associated with telomere maintenance, made exceptions for PLCB2 and ABCC8, whose homologues have so far only been reported in association with telomere length regulation in yeast.
